# Concentrations of pharmaceuticals and other micropollutants in groundwater downgradient from large on-site wastewater discharges

**DOI:** 10.1371/journal.pone.0206004

**Published:** 2018-11-07

**Authors:** Sarah M. Elliott, Melinda L. Erickson, Aliesha L. Krall, Byron A. Adams

**Affiliations:** 1 U.S. Geological Survey, Mounds View, Minnesota, United States of America; 2 Minnesota Pollution Control Agency, St. Paul, Minnesota, United States of America; Purdue University, UNITED STATES

## Abstract

Large subsurface treatment systems (LSTS) and rapid infiltration basins (RIB) are preferred onsite wastewater treatments compared to direct discharge of treated wastewater to streams and adjacent facilities. Discharge of these wastewater treatments may result in contaminant loading to aquifers that also serve as drinking water sources downgradient from the discharge site. Until recently, few studies have characterized the contribution of micropollutants (e.g. pharmaceuticals, fragrances, flame retardants, etc.) to receiving aquifers. We conducted a pilot project to characterize the occurrence of micropollutants in groundwater downgradient from 7 on-site treatment systems in Minnesota, USA: 5 community LSTS and 2 municipal RIB. One downgradient monitoring well was sampled three times at each facility over one year. Of 223 micropollutants analyzed, 35 were detected. Total sample concentrations ranged from 90 to 4,039 ng/L. Sulfamethoxazole (antibiotic) was detected in all samples at concentrations from 7 to 965 ng/L. Other pharmaceuticals (0.12–1,000 ng/L), organophosphorus flame retardants (10–500 ng/L), and other anthropogenic chemicals (4–775 ng/L) were also detected. The numbers and concentrations of micropollutants detected were inversely related to dissolved oxygen and depth to water. Ratios of pharmaceutical concentrations to human-health screening values were <0.10 for most samples. However, concentrations of carbamazepine and sulfamethoxazole exceeded screening values at two sites. Study results illustrate that large on-site wastewater systems designed to discharge to permeable soil or shallow groundwater effectively deliver pharmaceuticals and other micropollutants to groundwater aquifers and could contribute micropollutants to drinking water via water supply wells.

## Introduction

Wastewater from municipal sewage treatment plants and septic systems contains a variety of micropollutants, such as pharmaceuticals, personal care products, hormones, and other organic wastewater contaminants [1–10]. Although the vast majority of research on micropollutants has focused on wastewater treatment plant (WWTP) discharges to surface water [11], decentralized on-site wastewater treatment (septic) systems can contribute micropollutants to groundwater [2, 5, 7, 12, 13]. Septic systems represent a substantial share of wastewater treatment worldwide, but they may not treat waste as effectively as centralized WWTP because of poor placement, undetected failure, and anaerobic conditions that typically prevail in these systems [14, 15]. Additionally, septic systems and other WWTP are generally designed to reduce nutrient and pathogen loads, not to remove micropollutants [4, 14].

The effect of micropollutants from septic systems can be especially severe when the water table is shallow and surficial materials are highly permeable, such as sands and gravels. Surficial sand and gravel aquifers typically have high hydraulic conductivity, resulting in rapid infiltration rates, low horizontal dispersion, and little opportunity for natural attenuation resulting in plumes from septic systems in the shallow part of the aquifer [16]. In developed regions without centralized wastewater collection, treatment and off-site discharge, the density of domestic septic systems, and multi-household discharge of micropollutants to groundwater could affect ecological health via discharge to streams and could cause human exposures if groundwater is the primary or sole source of drinking water [4, 5]. Centralized larger-volume discharges from community on-site wastewater sources are poorly characterized and could cause similar problems [17, 18].

In Minnesota, there are more than 550,000 on-site wastewater treatment systems throughout the state. Most are unpermitted on-site wastewater treatment systems (10,000 mid-sized and 540,000 small), which require no effluent discharge reporting or groundwater monitoring [14]. However, 210 of Minnesota’s on-site wastewater treatment facilities are large enough to require permitting and monitoring. Of the 210 permitted facilities in Minnesota, 160 are large septic drainfields (large subsurface treatment systems, or LSTS) and 50 are rapid infiltration basins (RIB). The large LSTS each discharge >3.8 million liters of wastewater to soils and groundwater each year. Municipalities and other entities use RIB facilities, which are large earthen basins specifically designed to allow fast infiltration of wastewater into soil to recharge groundwater.

LSTS and RIB have been encouraged since 1978 in lieu of direct discharge to surface waters [19]. The Environmental Protection Agency’s most recent national estimate of the number of large septic systems was more than 350,000 large systems [20] and >20 million smaller systems [21, 22]. Worldwide, infiltration of wastewater has been documented as a significant source of micropollutants to groundwater [23,24]. Bremer and Harter’s [25] analysis showed that risk to domestic wells from septic wastewater was strongly influenced by system size, separation distance, and the hydraulic conductivity of the aquifer. In Minnesota, residential and community water supplies often rely on wells that are set within the same aquifer or a connected aquifer as the LSTS drainfields.

Groundwater monitoring for nutrients and pathogens is required at large (>38,000 liters/day) on-site wastewater treatment facilities in Minnesota. However, almost no testing has occurred to determine if on-site wastewater treatment in shallow surficial sand and gravel (vulnerable) aquifer settings may be affecting local groundwater resources with micropollutant loading. We conducted a pilot project to characterize the presence of micropollutants in shallow groundwater underlying on-site wastewater treatment in vulnerable aquifer settings. The goals of the study were to determine if: 1) micropollutants were detectable in groundwater downgradient from large on-site wastewater discharges, 2) detected concentrations were comparable to other micropollutant detections in the environment, 3) site-specific conditions and general water quality parameters relate to or influence micropollutant detections and concentrations, and 4) detected concentrations were near levels of concern for human health. This study provides a baseline of micropollutant data downgradient from seven large on-site wastewater systems located in vulnerable hydrogeologic settings. The ubiquity of on-site wastewater treatment systems and detection of micropollutants in groundwater is a concern for both well users and policymakers, which illustrates the international relevance of this issue.

## Materials and methods

Site access for this study was coordinated by the Minnesota Pollution Control Agency (MPCA). Because these wells are sampled regularly for conventional contaminants by the MPCA, no specific permits were required.

### Site descriptions

Seven on-site wastewater treatment facilities, 5 LSTS and 2 RIB, were selected for sampling based on conventional pollutant monitoring results and a range of facility characteristics, such as wastewater pretreatment and facility size (Table 1). All facilities have been in operation for ≥15 years. Pretreatment at the selected facilities varies and ranges from no pretreatment to textile filters, wetlands, or ponds. Six of the facilities had average annual effluent discharges ranging from 8 to 20.4 million liters. One facility (Facility F) had an annual average effluent discharge of 314.1 million liters. Drainfields for the community LSTS range in size from 0.3–0.9 hectares. Municipal RIB drainfields cover a range in area from 1.5 to 2.2 hectares.

**Table 1:** Large subsurface treatment systems (LSTS) and rapid infiltration basins (RIB) where groundwater was collected and analyzed for micropollutants, 2014–15.

Facility ID	Pretreatment	Annual design capacity discharge, ML	Average annual discharge (2013–15), ML	Drainfield or basin area, hectare	Approximate distance from edge of drainfield to monitoring well, meters	Community description	Monitoring well screen interval, meters BGS	Average water level, meters BGS
*LSTS*								
A	Constructed wetland	20.1	8.7	0.3	12	32 residential units	13.4–16.5	12.46
B	Textile filters, denitrification tank	35.9	19.3	0.3	21	70 residential units	12.2–15.2	12.39
C	None	46.9	14.8	0.4	20	46 residential units	9.1–12.2	10.16
D	Textile filters	55.2	20.4	0.9	15	220 mobile homes	11.0–14.0	12.09
E	None	20.8	15.9	0.2	10	80 mobile homes	4.6–7.6	4.44
*RIB*								
F	Screens, aeration ponds, secondary pond	745.8	314.1	2.2	50	Municipality of ~7,500	2.4–5.5	0.66
G	Stabilization ponds	61.7	7.9	1.5	100	Municipality of ~350	4.0–7.0	5.12

ML, million liters; BGS, below ground surface

**Fig 1:**
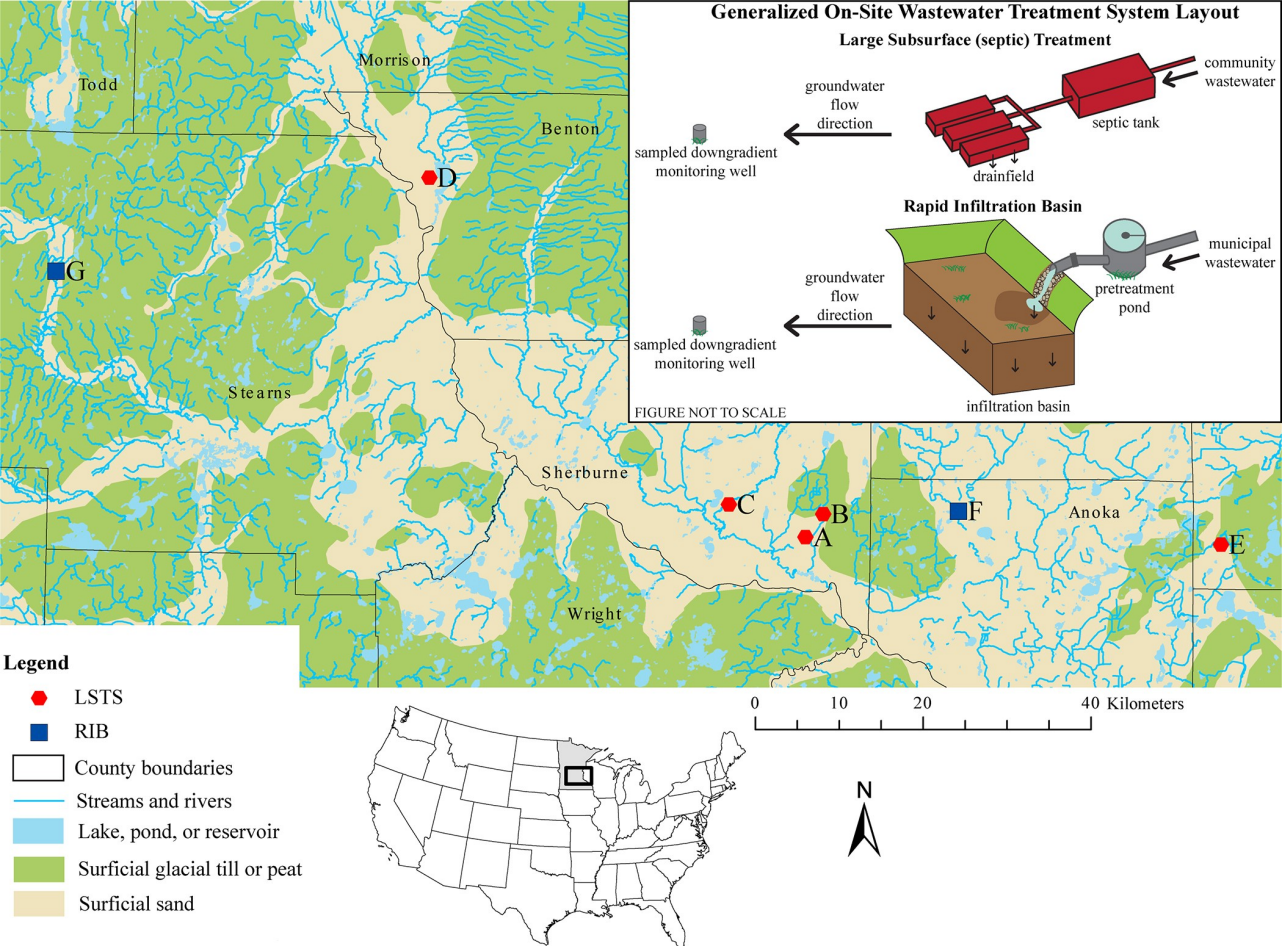
Location of on-site wastewater treatment facilities sampled in Central Minnesota, USA. Basemap from Hobbs and Goebel [26]. LSTS, large subsurface treatment system; RIB, rapid infiltration basin.

All facilities are located in hydrogeologic settings vulnerable to contamination from land surface as a result of highly permeable surficial materials, with a shallow (<15 meters) water table in sand and gravel aquifers (Fig 1). Past water level measurements in each facility’s monitoring wells were evaluated to determine groundwater flow direction, and one downgradient well at each facility was chosen for water quality sampling. Land use surrounding the facilities is variable, and includes residential development, undeveloped, and agricultural land for LSTS facilities and mixed urban development for the municipal RIB facilities.

Drinking water wells are <1 kilometer from the facilities included in this study [27]. Two residential developments have homes served by individual drinking water wells located 100–300 meters from the respective LSTS drainfield. Because of the limited scope and resources available, no drinking water wells were sampled as part of the study.

### Field sample collection methods

One downgradient monitoring well at each of the seven facilities was sampled in September 2014 (fall), May/June 2015 (spring), and August 2015 (summer). Field quality-assurance samples included two matrix-spike samples to characterize matrix interference with laboratory analyses and three blank samples to characterize potential contamination introduced during field activities.

Groundwater sample collection methods followed the USGS National Field Manual [28]. In summary, wells were purged using a submersible Grundfos or peristaltic pump outfitted with polytetrafluoroethylene tubing until three well volumes were removed and physical water-quality parameters [specific conductivity, pH, dissolved oxygen (DO), and temperature] stabilized. All samples were filtered through a 0.7 micron glass fiber filter, collected into baked amber glass or clear polyethylene bottles, and preserved according to method protocols. All samples were maintained at 4˚ C until received at the laboratories.

Sampling equipment was cleaned between wells using, in sequence, Liqui-Nox and tap water solution, tap water, deionized water, methanol, and organic-free blank water. Sampling personnel generally refrained from using personal-care products [e.g. mosquito repellant containing N,N-diethyl-meta-toluamide (DEET)] to avoid sample contamination during collection.

### Chemical analysis and reporting

Groundwater samples were filtered and analyzed for nutrients, ions, and boron at the Minnesota Department of Health Environmental Laboratory in St. Paul, Minnesota, using the following standard methods: ammonia-N, U.S. Environmental Protection Agency (EPA) 350.1 [29]; nitrate + nitrite-N, standard method (SM) SM 4500-NO3 F [30]; fluoride, SM 4500-F C [31]; bromide and chloride, EPA 300.1 [32]; and boron, EPA 200.7 [33].

Four different methods were used for analysis of 223 micropollutants. A total of 112 chemicals are included in the pharmaceutical method, which were determined by direct aqueous injection-high-performance liquid chromatography/tandem mass spectrometry [34]. The pharmaceutical method includes four chemicals that are not pharmaceuticals: atrazine, caffeine, nicotine, and methyl-1H-benzotriazole. These chemicals were counted as wastewater indicators for detection counts and concentration comparisons, leaving the total number of pharmaceuticals as 108. Fifty-nine wastewater indicator and other select chemicals were extracted through solid-phase extraction cartridges and then eluted from the cartridges with dichloromethane-diethyl ether and determined by capillary-column gas chromatography/mass spectrometry [35]. A total of 20 hormones and sterols were extracted from samples using solid phase extraction and analyzed by gas chromatography with tandem mass spectrometry [36]. Thirty-two antibiotics and select pharmaceuticals were extracted from samples using solid-phase extraction and determined by liquid chromatography/mass spectrometry [37]. Micropollutant data can be retrieved from the USGS National Water Information System (https://waterdata.usgs.gov/nwis) by searching for the station numbers provided in S2–S5 Tables.

The total number of individual micropollutants analyzed was 220 because caffeine, carbamazepine, and sulfamethoxazole are included in multiple analytical schedules. Compounds measured in a sample by multiple analytical methods were counted as one detection (not two) in summary tables and figures. The maximum concentration reported from any method was used in figures depicting concentrations.

### Quality assurance

Field quality-assurance samples collected for this study included two matrix-spike and three field equipment blank samples. Recovery of most chemicals in matrix-spike samples ranged from 70–120% (S1 Table). Recovery of several pharmaceuticals was relatively high, but none of those chemicals were detected in environmental samples. Conversely, recovery was relatively low (ranging from 50 to <70%) for several wastewater indicators. Those chemicals also were not detected in environmental samples. However, it should be noted that because of low recoveries, results may be biased low. Three chemicals were detected in field-equipment blank samples: 2-methylnaphthalene, 4-nonylphenol, and naphthalene. Nonylphenol was the only chemical detected above the reporting limit and which had a detection in environmental samples. The environmental detection was an estimated value below the reporting limit and blank-sample concentration and as a result, is not reported as a detection.

Laboratory quality-assurance samples included surrogate spikes, isotope dilution standards, and laboratory blank samples. Laboratory surrogate spike and isotope dilution standard results are provided with the analytical results in S2 through S4 Tables. Generally, recovery of surrogate spikes and isotope dilution standards was within 50 to 150%. One exception was decafluorobiphenyl, which had recoveries consistently <70%. Concentrations were not corrected for recoveries for this analysis. Three chemicals were potentially affected by laboratory contamination, as indicated by laboratory-blank sample data: nonylphenol, hexahydrohexamethyl cyclopentabenzopyran (HHCB), and glyburide. Associated data were qualified by the analyzing laboratory to indicate potential contamination. This was true for 6, 3, and 2 data points for nonylphenol, HHCB, and glyburide, respectively. These data were not included in data analyses.

### Comparison to human-health screening values

Screening values for 11 of the detected pharmaceuticals are available from the Minnesota Department of Health (Table 2). These values were established through a rapid assessment that does not consider full toxicological assessment and thus, are meant to be more conservative than traditional guidance values or standards [38]. To provide context for detected concentrations, screening value ratios were calculated by dividing detected concentrations by their respective screening value. Ratios of individual pharmaceuticals to screening values were classified as high if greater than the screening value, moderate if greater than 0.10 of the screening value, and low if less than 0.10 of the screening value. Total sample screening value ratios where calculated by summing individual screening value ratios for all detected pharmaceuticals in a given sample to provide an indication of overall potential hazard.

**Table 2:** Human-health screening values used to compare detected pharmaceutical concentrations in groundwater downgradient from large subsurface treatment systems and rapid infiltration basins.

Pharmaceutical	Screening value^a^, ng/L
Alprazolam	30
Carbamazepine	900
Carisoprodol	30,000
Fluconazole	400
Glyburide	4
Meprobamate	10,000
Metformin	4,000
Sulfamethoxazole	400
Temazepam	80
Tramadol	7,000
Warfarin	70

^a^Suchomel et al. [38]

## Results and discussion

**Fig 2:**
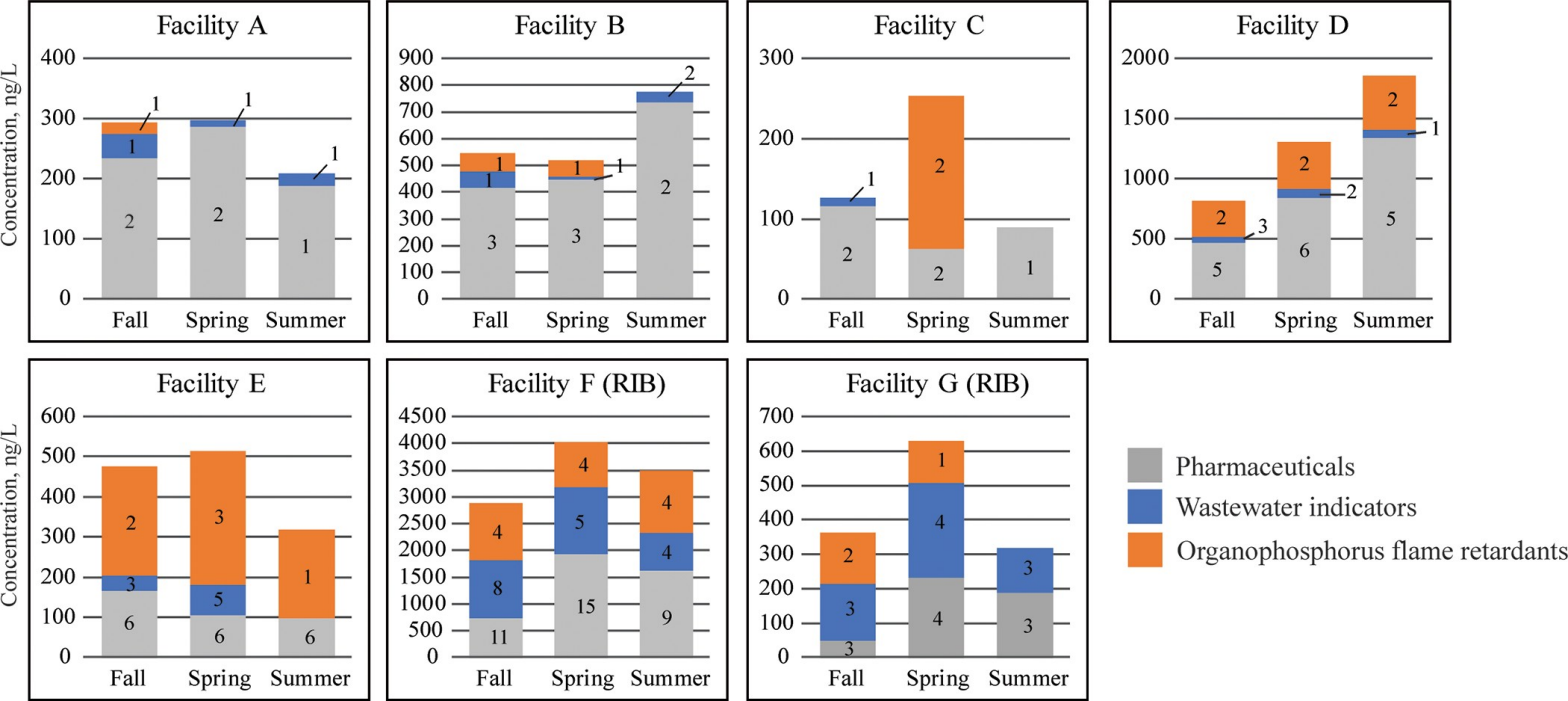
Total micropollutant concentrations detected in groundwater downgradient from large subsurface treatment systems and rapid infiltration basins. Numbers in bars indicate the number of micropollutants detected within that class.

Of the 223 analyzed, 35 unique micropollutants were detected in at least one sample: 18 pharmaceuticals and 17 wastewater indicators. In contrast to other studies of wastewater-affected groundwater [5, 17], no hormones were detected during this study. The number of micropollutants detected in samples ranged from 1 (Facility C, 8/11/2015) to 24 (Facility F, 5/28/2015) (S2–S5 Tables). The most micropollutants (24) and greatest total sample concentration (4,039 ng/L) were detected in groundwater downgradient from Facility F (Fig 2), the facility with the largest wastewater discharge. However, contrary to what might be expected, the fewest micropollutants and lowest concentrations were not observed at Facility G, the facility with the smallest wastewater discharge (Fig 2).

### Pharmaceuticals

The detected pharmaceuticals represent a variety of use classes with concentrations ranging from 0.12 (glyburide) to 1,000 (carbamazepine) ng/L (Table 3). Average total sample pharmaceutical concentrations ranged from 90 (Facility C) to 1,440 (Facility F) ng/L (S2 Table). Six pharmaceuticals were detected in ≥25% of samples. With few exceptions, individual pharmaceuticals were generally detected in multiple samples collected from the same facility and at similar concentrations, indicating a continual loading of pharmaceuticals. To further corroborate this, no consistent pattern in pharmaceutical detections compared to sample collection date was observed (Fig 2).

**Table 3:** Summary of micropollutant concentrations (ng/L) in groundwater samples collected downgradient from large subsurface treatment systems or rapid infiltration basins.

Chemical	RL	Percent detections (n = 21)	Percent detections above RL	Minimum	Median	Maximum
Alprazolam	21.3	14	0	0.62	1.32	2.41
Bupropion	17.8	29	0	2.82	7.72	27
Carbamazepine	11	71	62	9.93	38.4	1,000
Carisoprodol	12.5	10	0	12.4	12.8	13.2
Dehydronifedipine	24.5	14	0	8.96	10.6	14.3
Dextromethorphan	8.2	5	0	na	na	1.46
Diphenhydramine	5.79	5	0	na	na	6.19
Fluconazole	71	71	24	6.48	44.7	124
Glyburide	3.95	10	0	0.12	na	1.08
Lidocaine	15.2	43	24	0.83	39.1	76.9
Meprobamate	86	24	0	30.4	39.4	57.6
Metformin	13.1	14	14	75.6	91.3	206
Methocarbamol	8.72	5	5	na	na	550
Phenytoin	188	10	0	54.1	Na	115
Sulfamethoxazole	26.1	100	86	7	129	965
Temazepam	18.4	5	5	na	na	20.3
Tramadol	15.1	29	29	26.4	97.1	186
Warfarin	6.03	10	0	2.5	na	5.13
1,4-dichlorobenzene	40	10	0	18	na	20
Atrazine	19.4	29	14	7.59	21.5	47.3
Prometon	120	14	0	70	80	90
Tributyl phosphate	160	19	0	10	30	40
Triphenyl phosphate	120	10	0	20	na	30
Tris(2-butoxyethyl) phosphate	800	14	0	300	400	500
Tris(2-chloroethyl) phosphate	100	52	24	50	100	240
Tris(dichloroisopropyl) phosphate	160	57	43	110	240	440
Acetyl hexamethyl tetrahydro naphthalene (AHTN)	28	14	0	6	9	13
Caffeine	90.7	10	0	7.83	na	50
Hexahydrohexamethyl cyclopentabenzopyran (HHCB)	52	24	0	6	25	44
Isophorone	32	5	0	na	na	4
Methyl-1H-benzotriazole	141	43	14	14.7	67.1	775
N,N-diethyl-m-toluamide (DEET)	60	71	19	10	30	140
Nicotine	57.8	10	0	6.32	na	29.9
Tetrachloroethene	120	10	0	10	na	10

RL, reporting level; na, not applicable

Carbamazepine (anticonvulsant), fluconazole (antifungal), and sulfamethoxazole (antibiotic) were the most frequently detected pharmaceuticals. Carbamazepine and fluconazole were detected in 71% of samples; sulfamethoxazole was detected in all samples. Carbamazepine and sulfamethoxazole have been identified in other studies as good indicators of wastewater-influenced groundwater [11, 39] and our study results are consistent. Furthermore, sulfamethoxazole can persist for long distances downgradient of subsurface wastewater treatment systems and therefore is commonly detected in groundwater affected by on-site treatment systems [39, 40]. Bupropion (antidepressant), lidocaine (anesthetic), and tramadol (pain reliever) were detected in ≥25% of all samples with maximum concentrations of 27, 77, and 186 ng/L, respectively.

### Wastewater indicators

Detected wastewater indicators included corrosion inhibitors, pesticides, solvents, organophosphorus-based flame retardants (OPFR), fragrances, and stimulants. Concentrations ranged from 10 (tributyl phosphate) to 775 (methyl-1H-benzotriazole) ng/L (Table 3). Five wastewater indicators were detected in ≥25% of samples. DEET was the most frequently detected wastewater indicator, detected in 71% of samples and at least once at every facility. This is consistent with other studies, which have frequently detected DEET [24, 41]. Although DEET was frequently detected, concentrations generally were low (below the reporting level).

At least one OPFR was detected at all facilities (S3 Table). Two OPFRs were detected in ≥50% of samples and 5 facilities: tris(dichloro isopropyl) phosphate (TDIP) and tris(2-chloroethyl) phosphate (TCEP). Both OPFRs are used in upholstered furniture and other common household foam and fabric products. Concentrations of the individual OPFRs ranged from 10 (tributyl phosphate) to 500 [tris(2-butoxyethyl) phosphate] ng/L, with a median of 120 ng/L. In contrast, Schaider et al. [4] infrequently detected (≤25% of samples) OPFRs and at low concentrations (11 to 38 ng/L). The detection frequency and concentrations of TCEP and TDIP are consistent with low wastewater treatment removal efficiencies [42].

Several other micropollutants were detected in 5–43% of samples (Table 3). These included low concentrations (below the method reporting level) of musk fragrances [acetyl hexamethyl tetrahydro naphthalene (AHTN) and hexahydrohexamethyl cyclopentabenzopyran (HHCB)], industrial chemicals (isophorone and tetrachloroethene), and stimulants (nicotine and caffeine). The industrial chemical methyl-1H-benzotriazole (BTA) was detected in 43% of samples at concentrations ranging from 14.7 to 775 ng/L. The pesticides 1,4-dichlorobenzene (mothballs), atrazine (broadleaf herbicide), and prometon (non-selective herbicide) were detected in 10–29% of samples. Atrazine was detected only at Facilities F and G (both RIB) where it is used for weed control near holding ponds and infiltration basins.

### Comparison to detections in ambient groundwater, receiving surface waters, wastewater treatment plant effluents, and other on-site treatment systems

**Fig 3:**
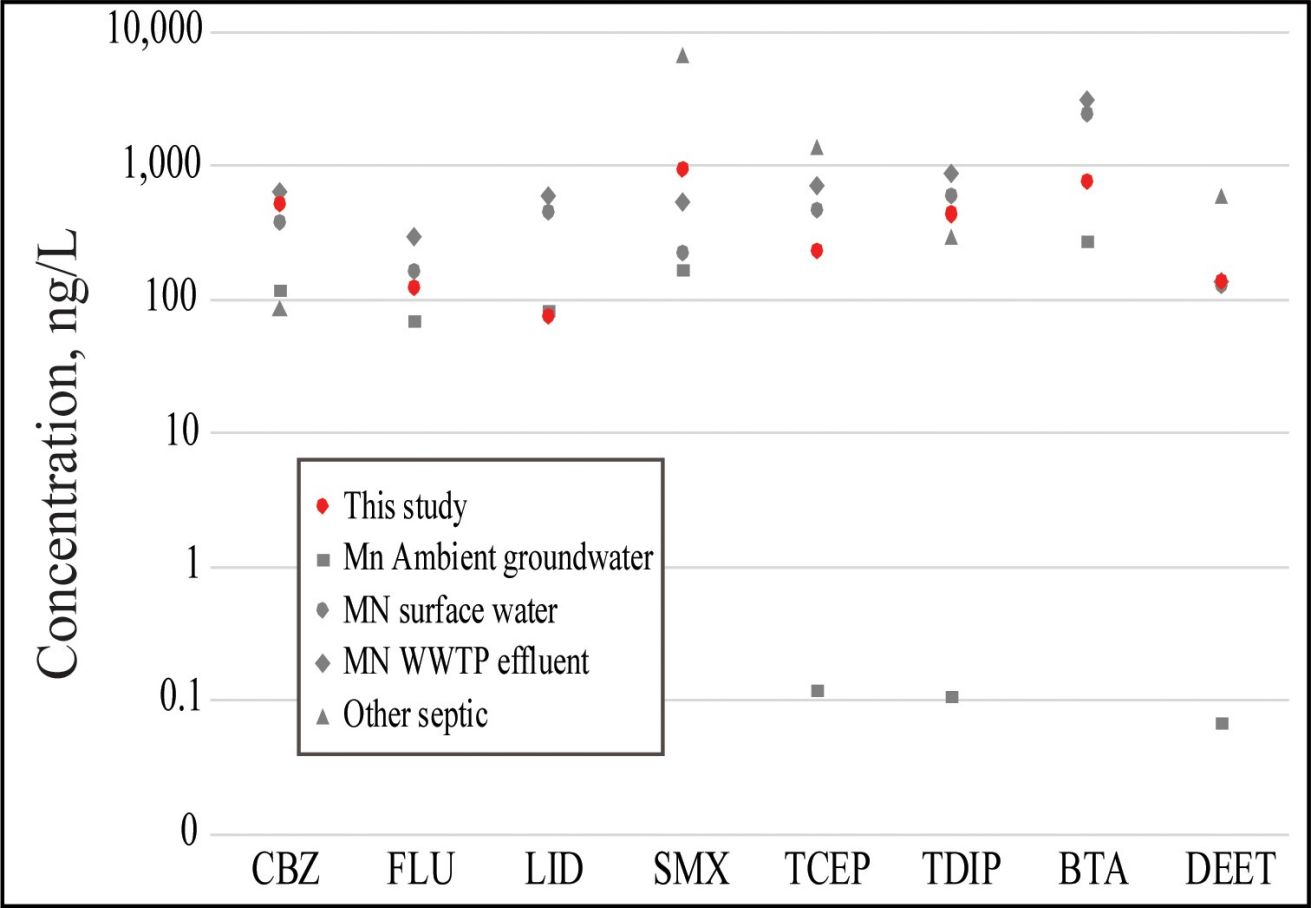
Comparison of maximum concentrations of most frequently detected (≥30%) micropollutants in this study and Minnesota (MN) ambient groundwater [9], MN surface water [6], MN wastewater treatment plant (WWTP) effluent [6], and other septic systems [7]. CBZ, carbamazepine; FLU, fluconazole; LID, lidocaine; SMX, sulfamethoxazole; TCEP, tris(2-chloroethyl) phosphate; TDIP, tris(dichloroisopropyl) phosphate; BTA, methyl-1H-benzotriazole; DEET, *N*,*N*-diethyl-meta-toluamide.

Micropollutants were detected more frequently and at higher concentrations in this study compared to ambient groundwater in Minnesota (S6 Table, Fig 3). Detection frequencies for the micropollutants in this study were >10% for 59% of detected chemicals, whereas detection frequencies in ambient groundwater were generally <5% [9]. Of the 15 micropollutants in common between the two studies, maximum concentrations of 7 (carbamazepine, fluconazole, sulfamethoxazole, triphenyl phosphate, methyl-1H-benzotriazole, DEET, and nicotine) were greater in our study compared to ambient groundwater (Fig 3; S6 Table). These results confirm that the sampled groundwaters in our study are under the influence of sources with chemical signatures indicative of wastewater.

Concentrations in this study were often higher than those detected in surface water, and some concentrations (e.g., carbamazepine, sulfamethoxazole, nicotine, and prometon) were similar to or higher than concentrations detected in wastewater effluent discharging directly to surface water (S6 Table, Fig 3). For example, concentrations of sulfamethoxazole in our study were 5.6, 4.3, and 1.8 times greater than ambient groundwater, receiving surface waters and wastewater effluents, respectively (S6 Table).

The number of micropollutants detected in our study was more similar to wastewater effluent from centralized municipal wastewater facilities. Several chemicals (alparazolam, dehydronifedipine, tramadol, and warfarin) were not detected in Minnesota wastewater effluents or receiving streams but were detected in our study (S6 Table) [6]. In a study of pharmaceuticals in Burlington, Vermont wastewater, several pharmaceuticals (alprazolam, dehydronifedipine, phenytoin, and warfarin) were not detected in wastewater effluent but were detected in our study. Additionally, higher concentrations of carbamazepine and SMX were found in our study’s groundwater compared to treated wastewater effluent [43]. Both carbamazepine and SMX were detected more frequently in our study, and the maximum detected concentrations were similar to Fairbairn et al. [44].

Schaider et al.’s [7] compilation of 20 studies reporting micropollutant concentrations associated with on-site septic system leachate included 13 of the micropollutants detected in this study. Our study detected two pharmaceuticals (diphenhydramine and warfarin) in groundwater that were not detected within septic system drainfields in Schaider et al [7]. Additionally, two micropollutants were detected in our study at higher concentrations (carbamazepine and TDIP). Results demonstrate that centralized larger-volume discharges from community on-site wastewater sources are important sources of micropollutants to groundwater. When the discharges are in proximity to drinking water wells, as is the case for our study’s facilities, drinking water quality is at risk.

### Micropollutant relations to conventional pollutants and other factors

The number, variety, and concentrations of detected pharmaceuticals and conventional pollutants illustrate that large on-site wastewater treatment systems integrate the pharmaceutical and other chemical usages of their large contributing populations (Tables 1 and 3; S2–S5 and S7 Tables). Large on-site wastewater systems designed to discharge to permeable soil or shallow groundwater effectively deliver pharmaceutical and other micropollutants to the groundwater system.

#### Conventional pollutants and redox

**Fig 4:**
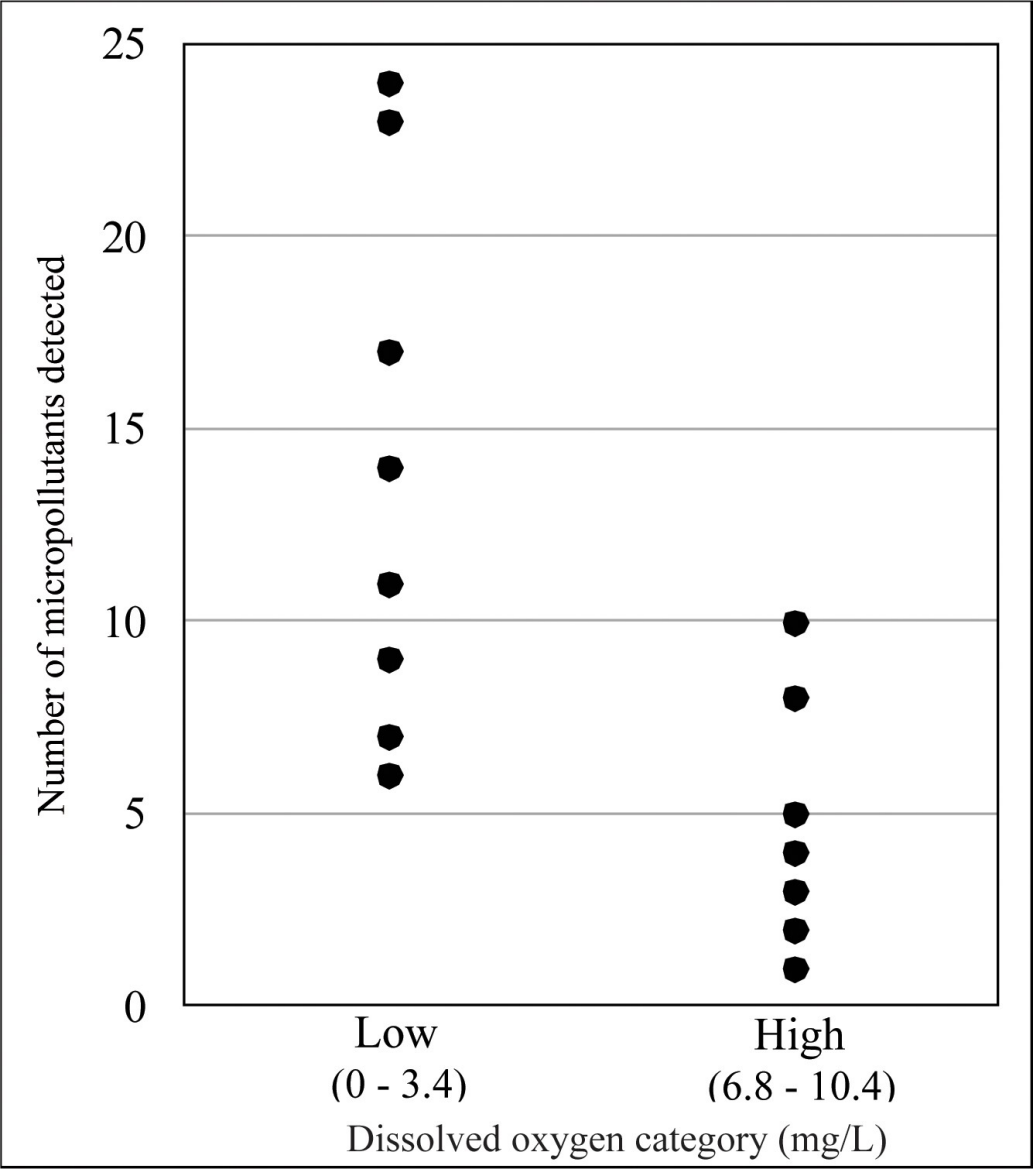
Number of micropollutants detected by dissolved oxygen category.

Many studies illustrate that micropollutants can be more resistant to removal in anoxic conditions when compared to oxic conditions [1, 5, 45]. Results from this study are consistent with the findings of others. More micropollutants were detected at facilities with less oxic or anoxic (<3.5 mg/L) groundwater conditions when compared to facilities with oxic (>6.5 mg/L) groundwater conditions. Up to 10 micropollutants were detected at facilities with oxic groundwater conditions, while up to 24 micropollutants were detected at facilities with less oxic or anoxic groundwater (Fig 4). The negative relationship between number of micropollutants detected and DO is significant (Spearman’s rank correlation, p<0.01). However, because Facility F is an outlier it drives the significance of the relationship. Although a generally negative relationship is still observed when removing data points from Facility F, the relationship is no longer significant (p = 0.08).

Facility F had a higher number of micropollutant detections (up to 24) and higher total micropollutant concentrations (3,498–4,039 ng/L) than smaller systems (Fig 2). This large system also had the lowest DO (0.2–0.5 mg/L, anoxic), relatively low (1.5 and 7.7 mg/L) nitrate-N in most samples, and detectable ammonia-N concentrations in two of three samples downgradient, despite a very shallow groundwater depth (approximately 0.6 meters below ground surface) (S7 Table). At the smaller facilities, DO ranged from 1.1–10.4 mg/L (sub-oxic to oxic) with groundwater depths ranging from approximately 4–13 meters below ground surface. Our results indicate that the high micropollutant concentrations and high detection frequencies observed in Facility F samples are related both to its large wastewater discharge volume and to the anoxic conditions in shallow downgradient groundwater.

#### Facility characteristics and season

The occurrence and total concentrations of micropollutants detected in our study were variable with annual discharge volume, groundwater depth, and downgradient redox conditions, which is consistent with other studies [24, 46]. Fewer detections of micropollutants at smaller facilities with oxic groundwater conditions indicate that smaller total loading and geochemical degradation processes diminish micropollutant transport downgradient in groundwater. Both carbamazepine and sulfamethoxazole have been found to be very persistent in groundwater and can be transported downgradient well away from the wastewater source discharge [39].

Slight seasonal differences in micropollutants were evident in this study. All wells had fewer micropollutants detected during the late summer (August) sampling event than during the early summer (May/June) or autumn (September) sampling events (Fig 2). Additionally, many wells had lower total concentrations of micropollutants in the late summer compared to early summer (Fig 2), when groundwater temperatures were generally higher than in the early summer. These micropollutant decreases may be due to increased biodegradation during the summer months.

### Potential human-health hazard

Domestic wells can be placed downgradient from and within the same aquifer receiving LSTS or RIB discharges. Although downgradient drinking water wells were not sampled as part of our pilot project, putting the data into context with regards to human-health hazards provides information that can be used to guide future research focusing on the fate and hazards of micropollutants in groundwater. Concentration data were compared to rapid assessment screening values established by the Minnesota Department of Health [38]. This analysis can also be used to prioritize micropollutants that may be of greater concern than others. Screening value ratios ranged from <0.01 to 2.4 for the 11 pharmaceuticals for which comparisons could be made (S8 Table). Of the 72 detected concentrations, 32 (44%) were moderate, and 3 (4%) were high, relative to screening values. Concentrations of meprobamate were consistently substantially lower (<0.01) than screening values in the five samples in which it was detected. A majority of the moderate concentrations were attributed to sulfamethoxazole at four of the seven facilities (S8 Table). Concentrations of carbamazepine were moderate in all samples collected from Facilities B and D, and high for one sample at Facility G (S8 Table). Average screening value ratios among all sites resulted in the following ranking of pharmaceuticals: sulfamethoxazole>carbamazepine>temazepam>fluconazole>tramadol. However, site-specific differences were observed. For example, with respect to average screening value ratios, tramadol was identified as the second most important pharmaceutical for LSTS sites, but fourth for RIB.

Human health screening values are often calculated or promulgated on a chemical-by-chemical basis. Although individual micropollutant concentrations detected in our study were generally below drinking water screening values, little is known in a quantitative way about the effects of mixtures. Summing the screening value ratios for every chemical detected within a given sample, provides a rough indication of the potential hazard associated with exposure to chemical mixtures. Although this method does not account for specific chemical interactions (e.g. additive, synergistic, antagonistic), it provides a method to prioritize sites based on a potential overall hazard with larger values representing more potential hazard. Total sample screening value ratios ranged from 0.038 (Facility E, September 2014) to 2.8 (Facility F, August 2015) (Table 4). The two facilities with higher annual discharge (Facilities D and F) had the highest average total sample screening value ratios (Table 4). However, volume of discharge and average total sample screening value ratios were not linearly related. For example, Facility G had the lowest discharge volume of the sampled sites, but the third highest average total sample screening value ratio. All but two samples (one from Facility E and one from Facility G) had a total sample screening value ratio exceeding 0.1, or moderate total concentration of organic constituents.

**Table 4:** Total sample screening value ratios for pharmaceuticals detected in groundwater downgradient from subsurface treatment systems in Central Minnesota, 2014–2015.

Facility	Average annual discharge, million liters per day	Date sampled	Total sample screening value ratio
A	8.7	September 3, 2014	0.59
		May 26, 2015	0.72
		August 10, 2015	0.47
		Average	0.59
B	19.3	September 3, 2014	0.60
		May 26, 2015	0.87
		August 10, 2015	0.70
		Average	0.72
C	14.8	September 3, 2014	0.29
		May 26, 2015	0.15
		August 10, 2015	0.11
		Average	0.18
D	20.4	September 4, 2014	0.61
		May 27, 2015	1.3
		August 11, 2015	1.2
		Average	1.04
E	15.9	September 5, 2014	0.038
		May 28, 2015	0.47
		August 13. 2015	0.44
		Average	0.32
F	314.1	September 5, 2014	0.84
		May 28, 2015	2.6
		August 12, 2015	2.8
		Average	2.1
G	7.9	September 4, 2014	0.09
		May 27, 2015	2.2
		August 11, 2015	0.33
		Average	0.87

The analyses provided herein provides context for environmental data related to human-health hazards and can help prioritize micropollutants and/or sites for further monitoring. However, there remains a lack of information regarding screening values, which could allow more rigorous evaluation of human health effects from more individual micropollutants, and more importantly, complex mixtures of micropollutants.

## Conclusions

We sampled groundwater downgradient from 7 of Minnesota’s approximately 210 large wastewater facilities discharging to shallow soil or groundwater. The number, variety, and concentrations of chemicals detected illustrates that large wastewater treatment systems integrate the pharmaceutical and other chemical usage of their large contributing populations and effectively deliver pharmaceuticals and other micropollutants to the groundwater system. Although the study results do not fully capture the flowpath of micropollutants in groundwater (no deeper aquifer wells, drinking water wells, or downgradient surface water was sampled), the results do show that on-site treatment facilities serving relatively small communities (hundreds to thousands of residents) result in numerous micropollutants in groundwater downgradient of on-site wastewater discharges. The presence of micropollutants in groundwater in sand and gravel aquifers in proximity to drinking water wells raises human-health concerns. But the lack of health-based drinking water standards and toxicological information limits quantification of risk. Micropollutant releases from large and small on-site wastewater treatment systems similar to those sampled in this study likely contribute micropollutants to surface waters or drinking water wells, a concern for both well users and policymakers worldwide. Further research focused on fate and transport of micropollutants to downgradient drinking water wells and surface waters is needed to fully understand the contribution of micropollutants to the environment from LSTS and RIB facilities.

## Supporting information

S1 Table Laboratory matrix-spike sample recoveries. (XLSX)

S2 Table Sample results for pharmaceuticals (laboratory method 2440). (XLSX)

S3 Table Sample results for wastewater indicators (laboratory method 1433). (XLSX)

S4 Table Sample results for hormones and sterols (laboratory method 2434). (XLSX)

S5 Table Sample results (ng/L) for antibiotics and other select pharmaceuticals (laboratory method LCAB). (XLSX)

S6 Table Comparison of maximum concentrations detected in groundwater downgradient from on-site wastewater discharges via large subsurface treatment systems or rapid infiltration basins and other studies. Concentrations in ng/L.
(XLSX)

S7 Table Summary of physical characteristics and inorganic chemical groundwater results from 21 groundwater samples collected downgradient from on-site wastewater discharges via large subsurface treatment systems or rapid infiltration basins, Central Minnesota. (XLSX)

S8 Table Ratios of detected concentrations to human health screening values [38] for 11 pharmaceuticals detected in groundwater samples downgradient from on-site wastewater discharges via large subsurface treatment systems or rapid infiltration basins, Central Minnesota. (XLSX)

## References

[1] Carrara C, Ptacek C, Robertson WD, Blowes DW, Moncur MC, Sverko E, et al 2008 Fate of pharmaceutical and trace organic compounds in three septic system plumes, Ontario, Canada. *Environmental Science &* *Technology* 42 (8): 2805–11. 10.1021/es070344q 18497127

[2] Conn KE, Barber LB, Brown GK, and Siegrist RL. 2006 Occurrence and fate of organic contaminants during onsite wastewater treatment. *Environmental Science & Technology* 40 (23): 7358–66. 10.1021/es0605117 17180989

[3] Ferrey, M. 2011. Wastewater treatment plant endocrine disrupting chemical monitoring study. Minnesota Pollution Control Agency, St. Paul, Minnesota. https://www.pca.state.mn.us/sites/default/files/lrp-ei-1sy11.pdf.

[4] Schaider LA, Ackerman JM, and Rudel RA. 2016 Septic systems as sources of organic wastewater compounds in domestic drinking water wells in a shallow sand and gravel aquifer. *Science of the Total Environment*. 547: 470–81. 10.1016/j.scitotenv.2015.12.081 26822473

[5] Swartz CH, Reddy S, Benotti MJ, Yin H, Barber LB, Brownawell BJ, et al 2006 Steroid estrogens, nonylphenol ethoxylate metabolites, and other wastewater contaminants in groundwater affected by a residential septic system on Cape Cod, MA. *Environmental Science & Technology* 40 (16): 4894–4902. 10.1021/es052595+ 16955883

[6] Elliott SM, Lee KE, Ziegeweid JR, Schoenfuss HL, and Martinovic-Weigelt D. 2016 Chemicals of emerging concern and fish biological endpoints data collected from select tributaries of the St. Croix River, Minnesota and Wisconsin, 2011–12: U.S. Geological Survey data release. 10.5066/F7M906RN

[7] Schaider LA, Rodgers KM, and Rudel RA. 2017 Review of organic wastewater compound concentrations and removal in onsite wastewater treatment systems. *Environmental Science & Technology*. 51(13):7304–17. 10.1021/acs.est.6b04778 28617596

[8] Valdés ME, Amé MV, Bistoni MA, and Wunderlin DA. 2014 Occurrence and bioaccumulation of pharmaceuticals in a fish species inhabiting the Suquía River Basin (Córdoba, Argentina). *Science of the Total Environment* 472: 389–96. 10.1016/j.scitotenv.2013.10.124 24295755

[9] Erickson, ML, Langer, SK, Roth, JL, and Kroening, SE. 2014. Contaminants of emerging concern in ambient groundwater in urbanized areas of Minnesota, 2009–12 (ver. 1.2, September 2014). U.S. Geological Survey Scientific Investigations Report 2014–5096, with appendix, 10.3133/sir20145096.

[10] Subedi B, Codru N, Dziewulski DM, Wilson LR, Xue J, Yun S, et al 2015 A pilot study on the assessment of trace organic contaminants including pharmaceuticals and personal care products from on-site wastewater treatment systems along Skaneateles Lake in New York State, USA. *Water Research*. 72(1): 28–39. 10.1016/j.watres.2014.10.049 25466637

[11] Barnes KK, Kolpin DW, Furlong ET, Zaugg SD, Meyer MT, and Barber LB. 2008 A national reconnaissance of pharmaceuticals and other organic wastewater contaminants in the United States—I) Groundwater. *The Science of the Total Environment* 402 (2–3): 192–200. 10.1016/j.scitotenv.2008.04.028 18556047

[12] Conn KE, Lowe KS, Drewes JE, Hoppe-Jones C, and Tucholke MB. 2010 Occurrence of pharmaceuticals and consumer product chemicals in raw wastewater and septic tank effluent from single-family homes. *Environmental Engineering Science* 27 (4): 347–56. 10.1089/ees.2009.0364

[13] Hinkle, SR, Weick, RJ, Johnson, JM, Cahill, JD, Smith, SG, and Rich, BJ. 2005. Organic wastewater compounds, pharmaceuticals, and coliphage in ground water receiving discharge from onsite wastewater treatment systems near La Pine, Oregon—Occurrence and implications for transport. U.S. Geological Survey Scientific Investigations Report 2005–5055. https://pubs.usgs.gov/sir/2005/5055/.

[14] Adams Byron. 2011 Best management practices and data needs for groundwater protection. Minnesota Pollution Control Agency, St. Paul, Minnesota.

[15] Ferrell G M, and Grimes BH. 2014 Effects of centralized and onsite wastewater treatment on the occurrence of traditional and emerging contaminants in streams. *Journal of Environmental Health* 76 (6): 18–27. 24645409

[16] Verstraeten IM, Fetterman GS, Meyer MT, Bullen T, and Sebree SK. 2005 Use of tracers and isotopes to evaluate vulnerability of water in domestic wells to septic waste. *Ground Water Monitoring and Remediation* 25 (2): 107–17. 10.1111/j.1745-6592.2005.0015.x

[17] Fisher IJ, Phillips PJ, Colella KM, Fisher SC, Tagliaferri T, Foreman WT, et al 2016 The impact of onsite wastewater disposal systems on groundwater in areas inundated by hurricane Sandy in New York and New Jersey. *Marine Pollution Bulletin* 107 (2): 509–17. 10.1016/j.marpolbul.2016.04.038 27261279

[18] Phillips P, Schubert C, Argue D, Fisher I, Furlong ET, Foreman W, et al 2015 Concentrations of hormones, pharmaceuticals and other micropollutants in groundwater affected by septic systems in New England and New York. *Science of the Total Environment*. 512–513: 43–54. 10.1016/j.scitotenv.2014.12.067 25613769

[19] USEPA. 1977. Clean Water Act of 1977 Amendments, US Code Title 33 Chapter 26. http://uscode.house.gov/browse/prelim@title33/chapter26&edition=prelim.

[20] USEPA. 1999. The Class V underground injection control study Volume 16 Aquifer Remediation Wells https://www.epa.gov/sites/production/files/2015-08/documents/classvstudy_volume16-aquiferremediation.pdf

[21] USEPA. 2012. Case studies of individual and clustered (decentralized) wastewater management programs. https://www.epa.gov/sites/production/files/2015-06/documents/decentralized-case-studies-2012.pdf.

[22] USEPA. 2013. Annual report 2013: Decentralized wastewater management program highlights. https://www.epa.gov/sites/production/files/2015-06/documents/scb_decent_ar_2013_final-508compliant.pdf.

[23] Díaz-Cruz MS and Barceló D. 2008 Trace organic chemicals contamination in ground water recharge. *Chemosphere* 72 (3): 333–42. 10.1016/j.chemosphere.2008.02.031 18378277

[24] Lapworth D J, Baran N, Stuart ME, and Ward RS. 2012 Emerging organic contaminants in groundwater: A review of sources, fate and occurrence. *Environmental Pollution* 163: 287–303. 10.1016/j.envpol.2011.12.034 22306910

[25] Bremer JE and Harter T. 2012 Domestic wells have high probability of pumping septic tank leachate. *Hydrology and Earth System Sciences* 16 (8): 2453–67. 10.5194/hess-16-2453-2012

[26] Hobbs, HC and Goebel, JE. 1982. S-01 Geologic map of Minnesota, Quaternary geology. Minnesota Geological Survey. Retrieved from the University of Minnesota Digital conservancy, http://hdl.handle.net/11299/60085.

[27] Minnesota Department of Health. 2017. Minnesota Well Index (MWI). http://www.health.state.mn.us/divs/eh/cwi/.

[28] USGS, variously dated. National field manual for the collection of water-quality data: U.S. Geological Survey Techniques of Water-Resources Investigations, Book 9, Chaps. A1-A9.

[29] USEPA. 1993. Method 350.1: Determination of ammonia nitrogen by semi-automated colorimetry, Revision 2.0. https://www.epa.gov/homeland-security-research/epa-method-3501-determination-ammonia-nitrogen-semi-automated-colorimetry.

[30] Standard Methods Online. 2000. SM 4500-NO3 F 21st ED. Standard methods online—Standard methods for the examination of water and wastewater. http://standardmethods.org/.

[31] Standard Methods Online. 1997. SM 4500-F-C 21st ED. Standard methods online—Standard methods for the examination of water and wastewater. http://standardmethods.org/.

[32] USEPA 1997. Method 300.1: Determination of inorganic anions in drinking water by ion chromatography, Revision 1.0. https://www.epa.gov/homeland-security-research/epa-method-3001-revision-10-determination-inorganic-anions-drinking-water.

[33] USEPA. 1994. Method 200.7: Determination of metals and trace elements in water and wastes by inductively coupled plasma-atomic emission spectrometry, Revision 4.4. https://www.epa.gov/homeland-security-research/method-2007-determination-metals-and-trace-elements-water-and-wastes.

[34] Furlong ET, Noriega MC, Kanagy CJ, Kanagy LK, Coffe LJ, and Burkhardt MR. 2014 Determination of human-use pharmaceuticals in filtered water by direct aqueous injection—high-performance liquid chromatography/tandem mass spectrometry. *U*.*S*. *Geological Survey Techniques and Methods 5-B10*, 49 10.3133/tm5B10

[35] Zaugg SD, Smith SG, and Schroeder MP. 2006 Determination of wastewater compounds in whole water by continuous liquid–liquid extraction and capillary-column gas chromatography/mass spectrometry: U.S. Geological Survey Techniques and Methods, Book 5, Chap. B4. In *Methods of the National Water Quality Laboratory*, 30 10.3133/tm5B4

[36] Foreman WT, Gray JL, ReVello RC, Lindley CE, Losche SA, and Barber LB. 2012 Determination of steroid hormones and related compounds in filtered and unfiltered water by solid-phase extraction, derivatization, and gas chromatography with tandem mass spectrometry: U.S. Geological Survey Techniques and Methods, Book 5, Chap. B9, 118 10.3133/tm5B9.

[37] Meyer MT, Lee EA, Ferrell GM, Bumgarner JE, and Verns J. 2007 Evaluation of offline tandem and online solid-phase extraction with liquid chromatography/electrospray ionization-mass spectrometry for analysis of antibiotics in ambient water and comparison to an independent method. *U*.*S*. *Geological Survey Scientific Investigations Report 2007–5021*, 28 10.3133/sir20075021

[38] Suchomel A, Goeden H, Dady J, and Shubat P. 2015 Pharmaceutical water screening values report Minnesota Department of Health, St. Paul, Minnesota 56 pg. http://www.health.state.mn.us/divs/eh/risk/guidance/dwec/pharmwaterrept.pdf

[39] Godfrey E, Woessner WW and Benotti MJ. 2007 Pharmaceuticals in on-site sewage effluent and ground water, Western Montana. *Ground Water* 45 (3): 263–71. 10.1111/j.1745-6584.2006.00288.x 17470115

[40] Barber LB, Keefe SH, LeBlanc DR, Bradley PM, Chapelle FH, Meyer MT, et al 2009 Fate of sulfamethoxazole, 4-nonylphenol, and 17beta-estradiol in groundwater contaminated by wastewater treatment plant effluent. *Environmental Science and Technology* 43 (13): 4843–50. 10.1021/es803292v 19673274

[41] Brausch JM and Rand GM. 2011 A review of personal care products in the aquatic environment: Environmental concentrations and toxicity. *Chemosphere* 82: 1518–32. 10.1016/j.chemosphere.2010.11.018 21185057

[42] Blum KM, Andersson PL, Renman G, Ahrens L, Gros M, Wiberg K, et al 2017 Non-target screening and prioritization of potentially persistent, bioaccumulating and toxic domestic wastewater contaminants and their removal in on-site and large-scale sewage treatment plants. *Science of the Total Environment* 575: 265–75. 10.1016/j.scitotenv.2016.09.135 27744155

[43] Vatovec C, Phillips P, Wagoner EV, Scott TM, and Furlong ET. 2016 Investigating dynamic sources of pharmaceuticals: Demographic and seasonal use are more important than down-the-drain disposal in wastewater effluent in a University city setting. *Science of the Total Environment* 572: 906–14. 10.1016/j.scitotenv.2016.07.199 27581107

[44] Fairbairn DJ, Karpuzcu ME, Arnold WA, Barber BL, Kaufenberg EF, Koskinen WC, et al 2016 Sources and transport of contaminants of emerging concern: A two-year study of occurrence and spatiotemporal variation in a mixed land use watershed. *Science of the Total Environment* 551–552: 605–13. 10.1016/j.scitotenv.2016.02.056 26897403

[45] Garcia SN, Clubbs RL, Stanley JK, Scheffe B, Yelderman JC, and Brooks BW. 2013 Comparative analysis of effluent water quality from a municipal treatment plant and two on-site wastewater treatment systems. *Chemosphere*. 10.1016/j.chemosphere.2013.03.007 23557723

[46] Li WC. 2014 Occurrence, sources, and fate of pharmaceuticals in aquatic environment and soil. *Environmental Pollution* 187: 193–201. 10.1016/j.envpol.2014.01.015 . 24521932

